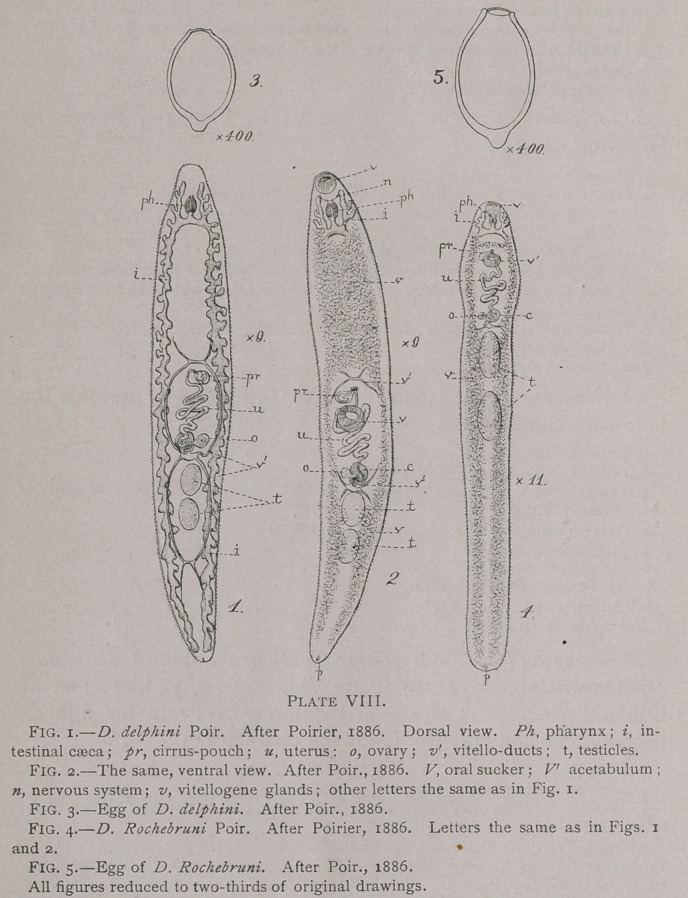# The Anatomy of the Large American Fluke (*Fasciola Magna*) and a Comparison with Other Species of the Genus Fasciola, S.ST.

**Published:** 1895-04

**Authors:** Chas. Wardell Stiles

**Affiliations:** Zoölogist, Bureau of Animal Industry


### THE ANATOMY OF THE LARGE AMERICAN FLUKE (*FASCIOLA MAGNA*) AND A COMPARISON WITH OTHER SPECIES OF THE GENUS FASCIOLA, S.ST.

BY CHAS. WARDELL STILES, PH.D.,
ZOÖLOGIST, BUREAU OF ANIMAL INDUSTRY.

### CONTAINING ALSO A LIST OF THE CHIEF EPIZOÖTICS OF FASCIOLIASIS (DISTOMATOSIS) AND A BIBLIOGRAPHY OF FASCIOLA HEPATICA.

BY ALBERT HASSALL, M.R.C.V.S.

(Continued from vol. xvi., 1895, page 147.)

COBBOLD ('79, pp. 393–400), in discussing the parasites of elephants, refers a number of times to *F. Jacksoni:*

The fifteen specimens possessed by Huxley, mentioned above, were removed from Burmese elephants in the summer of 1868 and sent to him from Rangoon, with a statement that they caused an extensive and fatal disease in Burmah; an historical review of this species up to 1879 is given; extracts from letters by General Hawkes (May 12, and July 30, 1875) are given regarding this parasite; as these extracts are very interesting, and to a certain degree important, it may be well to quote them:

"My attention has been recently directed to a very unusual mortality of elephants at this station. Out of twenty-eight elephants under my charge no less than twelve have died within the last sixteen months, whereas the average annual mortality has been hitherto only two per annum out of thirty-eight in our establishment. In every case of death there appeared to exist serious organic disease quite sufficient to account for such death, but as the mortality increased I had a post-mortem examination made in each case; and although here also organic disease sufficient to account for death was present in each case, yet in every one of these elephants we found liver-flukes in greater or less abundance. The only other published notice that I have been able to find of it is contained in a letter to a newspaper dated 'Rangoon, July 16, 1867, and is signed R. B.' In this letter the unusual mortality of seven elephants in about fifteen days is attributed to the presence of this liver-fluke, the two other parasites (*Amphistoma Hawkesii* and *Ascaris lonchoptera*) being also present in the intestines. Now in every case at which I was present *flukes were found in greater or less numbers* in the gall-ducts of the liver, and the *Amphistoma* was also as constantly present in the intestines, the soorti (*Asc. lonchoptera*), contrary to the general experience of the elephant attendants, being less frequently met with, though from its color and slender shape it is not so easily detected among the huge masses of fæces as the larger *Amphistoma*."

Both *Amphistoma* and *Fasciola Jacksoni* are reported in a case in the Secunderabad epizoöty. In a report of a later case by the veterinary surgeon (W. S. Adams) is found this statement:

I carried out the post-mortem examination with special reference to inquiry as to the probability of mortality among elephants at this station being of parasitic origin. This was suggested to me by the former case. The post-mortem appearances differed in every respect. There were flukes in the liver, but in no great quantity, and the structure of the liver was sound. Although not assisted by this case in attributing the mortality to parasitic origin, I am strengthened in my opinion that the death of the previous elephant was due to disease caused by the presence of the liver-fluke.

Von LINSTOW ('78, p. 45) records three species of liver-flukes (*D. hepaticum* Abildgaard, *D. elephantis* Jackson, and *D. Jacksonii* Cobbold) in *Elephas indicus*. Evidently the papers in which Cobbold stated that these recorded cases have reference to but one species have escaped his notice.

COBBOLD ('82, No. 11, pp. 242–246, Fig. 11, Pl. 24, Fig. 12) gives a new diagnosis of the species now under consideration:

" Body flat, orbicular, often folded toward the ventral surface, smooth to the naked eye, but armed throughout with numerous excessively minute dermal spines, which are larger above than below. Oral sucker terminal, small. Ventral sucker large and well forward. Reproductive papilla in the middle line, and placed considerably above the upper lip of the acetabulum; intromittent organ of great length. Digestive apparatus branched, its ramifications ending in cæcal terminations, which occupy nearly the whole extent of the body internally; œsophageal bulb distinct. Length, ½ to ⅝ of an inch; breadth, ⅓ to ½ of an inch. *Hab.* Biliary ducts and duodenum of *Elephas indicus*."

Cobbold again takes occasion, as he did many times before, to insist upon the priority of the generic term *Fasciola* for flukes with dendriform intestines and to the injustice of using the term *Distoma* for these flukes; he claims that spines are present in *F. Jacksoni;* they measure 1/700 to 1/750 of an inch long by 1/2000 of an inch thick (basal diameter); they are independent of the ridges described by Fitz, and are regularly arranged at equidistant points; in *F. hepatica* the spines are twice as long and three times as broad; the general dendriform arrangement is more or less similar in different specimens, but individual variation is naturally found; in specimens preserved in alcohol the greenish tint, due to bile, of the fresh specimen is not entirely destroyed; eggs are oval, 1/230 inch long by 1/330 inch broad, provided with a lid at one end; sexual openings in a depression, occasionally well marked, however, a papillary eminence can be distinguished in the centre of the depression.

BRAUN ('93, p. 910) places this species in the genus *Distomum*, sub-genus *Cladocœlium*, and remarks (p. 376) that Fitz has mistaken the vitellogene glands for the ovary. I cannot quite agree with my distinguished German colleague in this criticism.

Fitz evidently recognized the vitellogene glands, and he must also have seen the ovary. Whether he has also mistaken some other portion of the genital organs for a portion of the ovary is difficult to state, as his description is open to criticism, and as he fails to give figures.

### Specific Diagnosis.

*F. Jacksoni* Cobbold, 1869. Body, 12 to 16 mm. long by 8 to 12 mm. broad, flat, orbicular, often folded ventrally, smooth to naked eye, but armed throughout with minute spines, dorsal spines larger than ventral spines. Oral sucker terminal, small; ventral sucker large, well forward; genital papilla anterior to acetabulum; penis very long. Pharynx large, œsophagus (after C.'s figure) absent; intestinal cæca dendritic, median branches long. Other organs same as *F. hepatica.*

### V. The generic name Fasciola.

It is very generally admitted that the generic term *Fasciola* has priority over the term *Distomum*, and yet the latter term in some countries, at least, has entirely supplanted the former. In German literature of the present day *Fasciola* is hardly ever used. In French literature *Fasciola* is only occasionally met with. In English and American writings we find the term *Fasciola* more frequently than in the writings of other countries, yet not so frequently as the term *Distomum.* As I now take up the term *Fasciola*, it may be well to briefly review the generic history of the four flukes considered in this paper.

*Fasciola* was first proposed by Linné in 1746, and was also used in the tenth edition of the *Systema Naturæ* (1758). Linné included in the genus not only the liver-fluke, but also *Planaria* and *Schistocephalus.* The name *Distoma* Retzius was not proposed until 1786, and accordingly should not under any circumstances be allowed to supersede *Fasciola.* Goeze (1782) placed *F. hepatica* (the only species of the genus *Fasciola* s.st. known at the time) in his *Naturgeschichte* under the name *Planaria latiuscula.* Zeder reverted to *Distoma.* Dujardin[1] (1845) accepted the generic term *Distoma*, and proposed a division of the flukes into nine sub-genera. His first sub-genus (*Cladocœlium*) contained *D. hepaticum* as the type and only species, and was characterized by the ramified intestinal branches ("Intestin à

[1] Histoire Naturelle des Helminthes. Paris, 1845. *Vide* p. 388.

deux branches rameuses"), in contra-distinction to all other distomes known to him, the rest possessing simple intestinal cæca.

*Fasciola hepatica* continued, however, to be known under the generic terms *Fasciola*, *Distoma*, or *Distomum*, according to the individual tastes of the various authors. Blanchard and Cobbold upon several occcasions insisted upon the priority of the term *Fasciola* for flukes of the type of *F. hepatica* (*i. e.*, Dujardin's sub-genus *Cladocœlium*), and the latter, in 1858, published a *Synopsis of the Distomidæ*, in which the genus *Fasciola* is adopted for *F. hepatica* and *F. gigantea*, and in 1869 the species *F. Jacksoni* was added to it. As generic description of *Fasciola* Cobbold gives "the presence of a branched intestinal canal divided into numerous cæcal appendages." For the other distomes Cobbold created several genera, but few of them have been generally adopted, and they need not enter into the discussion here, for we are interested at the present moment only in flukes of the type of *F. hepatica*. Leuckart, in his *Parasiten des Menschen*, accepted *Fasciola* only as a sub-genus of the genus *Distomum*, giving as sub-generic diagnosis the following:

> "Body[1] of considerable size, broad and leaf-shape, provided with conically projecting anterior portion. The uterine loops are arranged almost like a rosette, posterior to the acetabulum. Intestine racemose and highly developed. The testicles are also racemose and highly developed, and occupy the entire middle field of the posterior half of the body."

Leuckart places in this sub-genus *D. hepaticum*, *D. Jacksoni* and *D. giganteum*.

Monticelli[2] then reverted to the sub-genus *Cladocœlium*, and quite recently Max Braun[3] adopted (evidently only provisionally) a modification of Dujardin's system, in which he admits eight of Dujardin's sub-genera, including under *Cladocœlium* ("Darm mit zwei verästelnden Schenkeln") *D. hepaticum* L., *D. giganteum* Cobbold, *D. Jacksonii* Cobbold, *D. magnum* Bassi, *D. delphini* Poir., *D. palliatum* Looss, *D. Rochebruni* Poir., and *D. oblongum* Cobd. He accepts the generic name "*Distomum* Retz, 1776," remarking (p. 894) that the name "*Fasciola* L. 1746, enthält

1 Körper von ansehnlicher Grösse, breit und blattförmig, mit einem zapfenartig vorspringenden Vordertheile. Die Uterus-windungen hinter dem Bauchsaugnapfe fast rosettenartig zusammengelegt. Darm verästelt und von mächtiger Entwicklung. Ebenso die Hoden, die mit ihren Zweigen das Mittelfeld der hintern Leibeshälfte vollständig ausfüllen.

2 Saggio di una morfologia dei Trematodi. Napoli, 1888. Unfortunately this paper is not at my disposal, but I quote from Blanchard and from Braun.

3 Vermes: Bronn's Klassen und Ordungen, etc., p. 909.

*Distomum*, *Planaria*, und *Schistocephalus* und ist nach Abtrennung der beiden letzten für die Distomen fast ganz ausser Gebrauch gekommen."

Stossich,[1] in a revision of distomes, raised the sub-genus *Cladocœlium* to generic rank, and included in it the species *C. hepaticum*, *C. elephantis*, *C. giganteum*, *C. delphini*, *C. palliatum*, and *C. Rochebruni*.

Summing up these statements the generic synonymy of flukes of the type *F. hepatica* would be as follows:

1746 et 1758, *Fasciola* Linné.
1782, *Planaria* Goeze.
1786, *Distoma* Retz.
1800, *Distoma* Zeder.
1809, *Distoma* Rud.
1845, *Distoma* (*Cladocœlium*) Duj.
1845, *Fasciolaria* (Anonymous).
1850, *Distomum* Dies.
1858, *Fasciola* (Linné, pars.) Cobbold.
1884, *Distomata* Taylor.
1888, *Distoma* (*Cladocœlium*) Monticelli.
1889, *Distomum* (*Fasciola*) R. Lkt.
1892, *Distomum* (*Cladocœlium*) Braun.
1892, *Cladocœlium* Stossich.

It is perfectly evident from the above that Retzius' name *Distoma* or *Stossisch's* generic (Dujardin's sub-generic) name *Cladocœlium* cannot be used instead of *Fasciola* without a total disregard for the law of priority. Accordingly, if the heterogeneous mass of distomes is included under one generic term, *Fasciola* must stand as the name of the genus. If the genus is divided (and this certainly must be done sooner or later), the genus with *F. hepatica* as type-species must receive the name *Fasciola*. Personally, I believe that flukes of the type of *F. hepatica* should be separated from the remaining distomes. The forms which authors have brought into close relationship with *F. hepatica* are: *F. gigantea*, *F. Jacksoni*, *F. magna*, *D. delphini* Poir., *D. palliatum* Looss, *D. Rochebruni* Poir., and *D. oblongum* Cobbold.

The forms *F. hepatica*, *F. gigantea*, *F. magna*, and *F. Jacksoni* agree in the following particulars:

[1] I Distomi dei Mammiferi; Estratto dal Programma della civica Scuola Reale superiore. Trieste, 1892.

16

1. The worms are hermaphrodites; body broad and flat.

2. The intestinal cæca are profusely branched.

3. The testicles are profusely branched and are situated on nearly the same height, for the greater part posterior to the transverse vitello-duct.

4. The ovary is branched and situated anterior to the transverse vitello-duct.

5. The vitellogene glands are enormously developed and occupy the margins of the entire worm, posterior to the acetabulum.

6. The uterus forms a rosette posterior to the acetabulum.

7. The genital pore is about half-way between the sucker and the acetabulum.

8. A cirrus is present.

*Hab.* Liver of various herbivorous animals.

PLATE VII.

*D. palliatum* Looss, 1884 (Plate VII., Fig. 1), presents the following characters:

1. Hermaphrodite; body narrow.
2. The intestinal cæca are but very slightly branched.
3. Testicles are lobate, one lying posterior to the other, both posterior to the transverse vitello-duct.
4. Ovary is slightly lobate and lies posterior to the transverse vitello-duct.
5. Vitellogene glands are not so profusely developed as in *F. hepatica*, etc., but extend some distance anterior to the acetabulum.
6. Uterus forms a rosette dorsally of the acetabulum.
7. Genital pore is only slightly anterior to the acetabulum.
8. Cirrus present.

*Hab.* Liver of *Delphinus delphis.*

*D. delphini* Poirier, 1886 (Plate VIII., Figs. 1–3) shows the following characters:

1. Hermaphrodite; narrow body.
2. Intestinal cæca are branched about as much as *D. palliatum.*
3. Testicles are ovoid, one posterior to the other, both posterior to the transverse vitello-duct.
4. Ovary spherical, anterior to transverse vitello-duct.
5. Vitellogene glands profusely developed, and extend anterior to acetabulum.
6. Uterus does not form a rosette (Cf. Poirier's figure), and is situated for the greater part posterior to acetabulum.
7. Genital pore is only slightly anterior to the acetabulum.
8. Cirrus evidently present.

*Hab.* Liver of *Delphinus delphis.*

*D. Rochebruni* Poir., 1886 (Plate VIII., Figs. 4, 5) presents the following characters:

1. Hermaphrodite; narrow.
2. Intestinal cæca same as in *D. delphini.*
3. Testicles ovoid, one posterior to the other, both in anterior half of body posterior to transverse vitello-duct.
4. Ovary spherical, anterior to transverse vitello-duct.
5. Vitellogene glands as in *D. delphini.*
6. Uterus as in *D. delphini.*
7. Genital pore as in *D. delphini.*
8. Cirrus present.

*Hab.* Liver of *Delphinus delphis.*

*D. oblongum* (Cobbold) (*Campula oblonga* Cobbold, *Distomum campanula* Cobbold, 1876) (Plate VII., Figs. 2, 3), like most of Cobbold's species, is very poorly described. From his figures, however, we can take the following characters:

1. Hermaphrodite; narrow.
2. Intestinal cæca zigzag in form, but not dendritic.
3. Testicles round to oval, one posterior to the other, both in posterior portion of the body, posterior to transverse vitello-duct.
4. Ovary. ? (From Cobbold's figures it is impossible to tell with certainty which of two organs drawn is the ovary, one of them is nearly spherical and lies anterior to the transverse vitello-duct, the other (probably this one is the receptaculum seminis) is somewhat oval and lies posterior to the vitello-duct.)
5. Vitellogene glands?

6. Uterus long, extends anterior and posterior to ovary: does not form a rosette.
7. Genital pore immediately anterior to acetabulum.
8. Cirrus ?

For this fluke, which Cobbold considered intermediate between *Fasciola* and *Distoma*, he proposed the generic term *Campula*, but he afterward rejected the genus. It seems to be a *Dicrocœlium*, second section.

From the above analysis of characters, I think it perfectly evident that *D. oblongum* is too insufficiently known to enter into consideration. Looking at the other forms we find that they represent two distinct groups, one containing *F. hepatica*, *F. gigantea*, *F. magna*, and *F. Jacksoni*, the other containing *D. palliatum*, *D. delphini*, and *D. Rochebruni*.

The only important character common to these two groups (aside from the hermaphroditism and presence of a cirrus) is the branching of the intestinal cæca, these being *profusely* branched in the first group, and but *slightly* branched in the second group. They differ from each other in the following characters:

| | First Group. | Second Group. |
|---|---|---|
| Ovary . . . . . | End at or very near the acetabulum. | Extend far anterior to the acetabulum. |
| Testicles . . . . | Profusely branched, may be side by side, or nearly so. | Ovoid to lobate; one posterior to the other. |
| Vitellogene glands | Branched. | Spherical to lobate. |

These correlated differences seem to me ample grounds for not uniting flukes of the type of *F. hepatica* with flukes of the second group. Certainly the branching of the intestines could easily occur as a parallelism in different genera, and the very fact that the branching is so much slighter in the second group than in the first, coupled with the other divergent characters, seems to indicate that the branching in *D. palliatum* is probably of more recent origin in this group than in *F. hepatica;* if that is true, then the second group is of entirely different origin from the first, and hence not generically related. If these seven flukes, however, are placed in the genus *Fasciola*, we must not forget that the genus is practically established upon a single character, and is very artificial. The first four flukes mentioned, on the other hand, agree in more than one important character, and represent a very compact and natural group; this fact, I believe, justified Cobbold in defining the genus *Fasciola* as he did.

If, on the other hand, we are to unite other flukes in this genus, then *D. crassum* must receive our attention, for that certainly resembles *F. hepatica* as much as do *D. palliatum*, *D. delphini*, and *D. Rochebruni*.

While agreeing thoroughly with Braun that the time has not yet come to establish definite genera for all the different forms included under the collective generic term *Distomum*, I consider that when we find a distinct group which contains several evidently very closely allied species differing very materially from the other forms—as in the case of the four species mentioned in this paper—it is best to recognize them as a distinct genus.

*(To be Continued.)*

## Figures and Tables

**Plate VII. f1:**
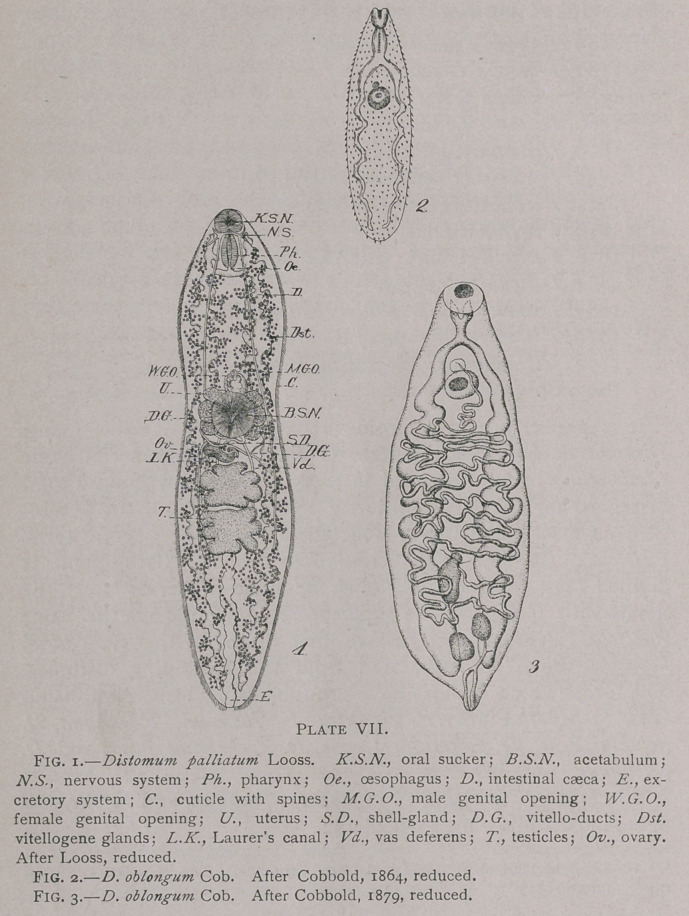


**Plate VIII. f2:**